# Supplementary material for: Digital health app development standards: a systematic review protocol
Source: BMJ Open. 2018 Aug 17;8(8):e022969. doi: 10.1136/bmjopen-2018-022969 (PMC6104800; doi:10.1136/bmjopen-2018-022969)
Supplement: Supplementary file 1 [file bmjopen-2018-022969supp001.pdf]

Supplementary File

Draft preliminary search

Regulatory website (International Organization for Standardization [ISO]) search terms (with limited search functions) in the technical sector of health (to be adapted for other websites)

1. Health app
2. Medical app
3. Health software
4. Medical software
5. Medical device
6. Health Information Technology System
7. Medical Information Technology System
8. Health IT system
9. Medical IT system

MEDLINE search strategy (to be adapted for other databases)

Health apps terms combined with OR:

Medical device*.ti,ab,kw.
Health app*.ti,ab,kw.
Medical app*.ti,ab,kw.
Wearable*.ti,ab,kw.
Mobile Applications/
Medical Informatics Applications/

Standards terms combined with OR:

(Standard* or framework* or Guideline* or Guidance or best practice* or Risk Assessment* or road map* or roadmap*) adj3 (Medical device* OR Health app* OR Medical app* OR Wearable*).ti,ab,kw.
Medical Device Legislation/st, td [Standards, Trends]
Device Approval/lj, st [Legislation & Jurisprudence, Standards]
Practice Guideline/
Risk Assessment/lj, mt, st [Legislation & Jurisprudence, Methods, Standards]
Government Regulation/

Final result = 1 AND 2

No limitations to be applied to the search strategy

Draft data extraction sheet

Items	Data	RIGHT checklist item[20]
Basic information		
Title/subtitle		1a
Year of publication		1b
Focus		1c
Scope		
Category (health app, medical device, software for medicine)		
Executive summary		2
Abbreviations and acronyms		3
Definitions		
Corresponding author/developer		4
Organisation		
Background		
Description of the problem		5
Aim		6
Primary population		7a
Subgroups		7b
Intended primary users		8a
Setting for intended use		8b
Document development group contributors		9a
Individuals involved in developing document		9b

Evidence		
Key questions		10a
Selection of outcomes		10b
Systematic review undertaken or used		11a
If based on existing systematic review, description of identification and assessment (provide the search strategies and the selection criteria, and description of how the risk of bias was evaluated) and whether they were updated		11b
Approach used for assessing evidence		12
Mention of other standards, guidelines, frameworks, and best practices		
Recommendations/requirements		
Clear, precise, and actionable recommendations/requirements		13a
Evidence for important subgroups		13b
Strength of recommendations/requirements		13c
Rationale/explanation for recommendations/requirements of target populations		14a
Cost and resource implication for recommendations/requirements		14b
Other factors taken into consideration when formulating the recommendations, such as equity, feasibility and acceptability		14c
Evidence to decision process		15
Review and quality assurance		
External review		16
Quality assurance		17

Funding, declaration and management of interest		
Sources of funding for all stages of document development		18a
Role of funder(s) in the different stages of document development and in the dissemination and implementation of the recommendations		18b
Types of conflicts (financial and non-financial)		19a
How conflicts of interest were evaluated and managed		19b
Other information		
Access		20
Suggestions for further research		21
Limitations of document		22
Comments		
