# Supplementary material for: Digital health app development standards: a systematic review protocol
Source: BMJ Open. 2018 Aug 17;8(8):e022969. doi: 10.1136/bmjopen-2018-022969 (PMC6104800; doi:10.1136/bmjopen-2018-022969)
Supplement: Author's manuscript [file bmjopen-2018-022969.draft_revisions.pdf]

BMJ Open is committed to open peer review. As part of this commitment we make the peer review history of every article we publish publicly available.

When an article is published we post the peer reviewers' comments and the authors' responses online. We also post the versions of the paper that were used during peer review. These are the versions that the peer review comments apply to.

The versions of the paper that follow are the versions that were submitted during the peer review process. They are not the versions of record or the final published versions. They should not be cited or distributed as the published version of this manuscript.

BMJ Open is an open access journal and the full, final, typeset and author-corrected version of record of the manuscript is available on our site with no access controls, subscription charges or pay-per-view fees (http://bmjopen.bmj.com).

If you have any questions on BMJ Open's open peer review process please email info.bmjopen@bmj.com

BMJ Open

Digital health app development standards: a systematic review protocol

Journal:	*BMJ Open*
Manuscript ID	bmjopen-2018-022969
Article Type:	Protocol
Date Submitted by the Author:	15-Mar-2018
Complete List of Authors:	Van Velthoven, Michelle; University of Oxford, Department of Paediatrics<br>Smith, James; University of Oxford, The Oxford – UCL Centre for the Advancement of Sustainable Medical Innovation (CASMI) Oxford; University of Oxford, Nuffield Department of Orthopaedics, Rheumatology and Musculoskeletal Sciences<br>Wells, Glenn; Oxford Academic Health Science Centre<br>Brindley, David; University of Oxford, Department of Paediatrics
Keywords:	Health & safety < HEALTH SERVICES ADMINISTRATION & MANAGEMENT, Health policy < HEALTH SERVICES ADMINISTRATION & MANAGEMENT, Protocols & guidelines < HEALTH SERVICES ADMINISTRATION & MANAGEMENT, Quality in health care < HEALTH SERVICES ADMINISTRATION & MANAGEMENT, Telemedicine < BIOTECHNOLOGY & BIOINFORMATICS

SCHOLARONE™
Manuscripts

For peer review only

Digital health app development standards: a systematic review protocol

Michelle Helena van Velthoven[1], James Smith[2,3], Glenn Wells[4], David Brindley[1]

[1]Healthcare Translation Research Group, Department of Paediatrics, University of Oxford, Oxford, UK
[2]The Oxford – UCL Centre for the Advancement of Sustainable Medical Innovation (CASMI), The University of Oxford, New Richards Building, Old Road Campus, Oxford, OX3 7LG, UK
[3]Nuffield Department of Orthopaedics, Rheumatology and Musculoskeletal Sciences, University of Oxford, Old Road, Oxford, OX3 7LD, UK.
[4]Oxford Academic Health Sciences Centre, Oxford, UK

Corresponding author:
Michelle Helena van Velthoven
Michelle.vanvelthoven@paediatrics.ox.ac.uk
Mobile: 07425167828

Word count: 2268

For peer review only

Abstract (word count: 289)

Introduction

There is currently a lack of clear and accepted standards for the development (planning, requirement analysis and research, design and application testing) of apps for medical and healthcare use, which poses different risks to developers, providers, patients and the public. The aim of this work is to provide an overview of the current standards, frameworks, best practices and guidelines for the development of digital health apps. This review is a critical 'stepping stone' for further work on producing appropriate standards that can help mitigate risks.

Methods and analysis

A systematic review identifying criteria from applicable standards, guidelines, frameworks, and best practices for the development of health apps. We will draw from standards for software for medical devices, clinical information systems, and medicine because of their relatedness and hope to apply lessons learnt to apps. We will exclude other types of publications, and those published in languages other than English. We will search websites of relevant regulatory and professionals organisations. For health apps, we will also search electronic research databases (e.g. MEDLINE, EMBASE, SCOPUS, ProQuest Technology Collection and Engineering Index) because relevant publications may not be found on other websites. We will hand-search reference lists of included publications. The review will focus on international, US, European, and UK standards because these are the markets of primary interest to the majority of app developers currently. We will provide a narrative overview of findings and tabular summaries of extracted data. Also, we will analyze the relationship between different standards and compare US and EU standards.

Ethics and dissemination

No ethics approval is required. The review will be disseminated through peer-reviewed publications, conference presentations, and inform efforts that aim to improve the quality of health apps through existing links with relevant organisations.

Key words: digital health, health apps, medical device, clinical software, medication

For peer review only - http://bmjopen.bmj.com/site/about/guidelines.xhtml

Strengths and limitations of this study

- This review will provide a systematic overview of standards for the development of health apps based on those for software of medical devices, clinical information systems, and medication given their relatedness.
- A comprehensive search of standards will be conducted.
- A limitation of this review is that it only focuses on standards reported by international organisations and those in the US, EU, and UK.
- The review will inform efforts that aim to improve the quality of health apps and is a critical 'stepping stone' to producing actionable guidelines for developers and adopters.

Introduction

Description of the issue

There is a lot of 'apptimism' for the potential of health apps to improve the quality of care and reduce costs.[1] However, despite a rapid growth of the health apps market with an estimated 325,000 health apps available in 2017[2] this potential has not been achieved. Health apps are software programs that are used in the context of healthcare on mobile communication devices, such as smartphones and tablets, that can also be used as accessories, such as wearable devices, or as a combination of accessories and software.[3] However, there are many low-quality and unsafe health apps and even apps with potentially harmful content.[4] This situation is resulting in different types of risks for users such as embarrassment, stigma, discrimination, stress, dissatisfaction, delay in effective treatment, poor lifestyle choices and deterioration in health.[5] Also, providers can be negatively impacted by reputation loss, poor quality of care, increase in undue demand on services, and opportunity losses.[5]

One of the reasons for the large number of low-quality health apps is that there are no agreed standards for their development, assessment, and appraisal. Health apps can be developed quickly, at any place and time by anyone interested, including people with non-medical backgrounds, which can create conflicting views on rapid technology development versus thorough evidence-based medicine principles.[6] Apps are often developed by start-ups with limited resources for research and development, which may result in short duration pilots with small participant numbers. Traditional healthcare companies with larger financial resources, such as pharmaceutical companies, on the other hand, have realized that they need to engage with digital health but are struggling given the differences between the development of drugs and digital tools.[7] As a result, there is a lack of consistency in the development of health apps.

Description of standards

A standard can be defined as "a document that provides requirements, specifications, guidelines or characteristics that can be used consistently to ensure that materials, products, processes, and services are fit for their purpose".[8] Standards are collaborative efforts, written by committees of manufacturers, users, research organizations, government departments and consumers.

For peer review only - http://bmjopen.bmj.com/site/about/guidelines.xhtml

Medical devices, clinical software and medicines have many standards, regulations and guidances for their development.[9] For example, the International Organization for Standardization (ISO) has a standard on software for medical devices, IEC 62304:2006 ‘ Medical device software - Software life cycle processes’, which complements the main standard for medical devices, ISO 13485:2016 ‘Medical devices - Quality management systems - Requirements for regulatory purposes’ and ISO 14971:2007, ‘Application of risk management to medical devices’.[10] Similarly, in pharmaceutical manufacturing, standards exist such as International Society for Pharmaceutical Engineering (ISPE) Good Automated Manufacturing Practice (GAMP) for computerized systems[11], and are widely adopted.

However, for health software development there is the concern that standards will inhibit innovation. There needs to be a balance between basic principles for safe and efficient development of health apps that allows products to be built correctly and efficiently. Efforts have been made to develop more proportionate and adaptive governance of innovative technologies for different types of innovation, in different industries sectors.[12]

The benefits of standards for health apps

Standards can mitigate the risks of health apps, including clinical, privacy and economic risks, which are influenced by the function(s) of the health app, user and contextual factors.[5] Health apps are clinical software and can be divided into higher-risk apps classified as medical devices, such as clinical-decision-support apps, and lower-risk apps that are not, such as wellness and fitness apps.

Standards can help with developing appropriate products that are fit for purpose. Standards can have economic benefits such as contributing to the growth of economies, productivity and GDP, and exports.[13] For companies, using standards can also enhance their reputation; improve compliance with regulations; and encourage innovation through the diffusion of knowledge. For users, standards can ensure the safety, quality, and consistency of products.[13]

Why it is important to do this review

Previous efforts have developed standards for certain health apps, such as the British Standards Institute (BSI) PASS 277:2015, a standard for quality criteria for health and wellness apps across the life cycle[14] which builds on more established approaches for clinical software such as the Association for the Advancement of Medical Instrumentation (AAMI) TIR45:2012 guidance on the use of agile practices in the development of medical device software.[15] However, such guidance is focused specifically on the UK, and there is a clear need to provide an overview of standards applicable to all health apps across broader jurisdictions. Additionally, understanding and collating the requirements for software development in closely related fields would be useful in informing development of standards at a later date. We will conduct a systematic review to address these needs.

For peer review only - http://bmjopen.bmj.com/site/about/guidelines.xhtml

Objectives

This systematic review is part of a larger project that addresses the current lack of clear standards for apps for medical and healthcare use and the risk that not having these standards poses to developers, providers, patients and the public. The objectives of this systematic review are to:

1. Provide an overview of currently applicable standards, guidelines, frameworks, and best practices relevant for the development of digital health apps;
2. Look at other not directly applicable but related standards to see if relevant lessons can be learned from current software-specific guidance for medical devices, medication, and clinical information systems.

The review will inform efforts that aim to improve the quality of health apps and is a critical 'stepping stone' to further research on producing actionable guidelines for developers and adopters.

Methods and analysis

This is the protocol for a systematic review that is reported where possible according to the Preferred Reporting Items for Systematic Reviews and Meta-Analyses for protocols (PRISMA-P),[16] which is provided as a supporting document.

Criteria for considering publications

We will include applicable standards, guidelines, frameworks, and best practices for the development (planning, requirement analysis and research, design and application testing[14]) of health apps. We will draw from software standards for medical devices, clinical information systems, and medicine because of their relatedness and apply hope to apply lessons learnt to apps.

Standards are requirements, specifications, guidelines or characteristics that can be used consistently to ensure that materials, products, processes, and services are fit for their purpose. Guidelines are advice or information aimed at resolving a problem or difficulty while frameworks are underlying structures for describing a process. A framework is 'a platform for developing software applications. It provides a foundation on which software developers can build programs for a specific platform.'[17] Best practice is a method or technique that has been generally accepted as superior to any alternatives.

An app is defined similarly by different organisations,[3,18] for example by the US Food and Drug Administration (FDA) as "software programs that run on smartphones and other mobile communication devices. They can also be accessories that attach to a smartphone or other mobile communication devices, or a combination of accessories and software".[3] In the context of healthcare, the FDA defines mobile medical apps as 'medical devices that are mobile apps, meet the definition of a medical device and are an accessory to a regulated medical device or transform a mobile platform into a regulated medical device'.[3] The Medicines and Health Regulatory Authority (MHRA) broadly considers health apps to be medical devices if they have a medical purpose (e.g. prevention, diagnosis, monitoring,

For peer review only - http://bmjopen.bmj.com/site/about/guidelines.xhtml

treatment of disease, diagnosis of disease, injury or handicap, compensation for injury or handicap, investigation, replacement of modification of the anatomy or of a physiological process, control of conception).[18] The BSI considers a health or wellness app when it ‘contributes to any aspect of the physical, mental or social wellbeing of the user or any other subject of care or wellbeing’.[14]

We will exclude other types of papers, such as editorials, opinion pieces, viewpoints, and publications in languages other than English. It will not be possible to provide an overview of standards in all countries around the world given our limited resources. Therefore, we will focus on international, US, European, and UK standards because these are the markets of primary interest to the majority of app developers currently.

Information sources

We will search the following standards databases for health apps, medical devices, clinical software, and medicines advised by Imperial College London librarians[19]:
-International Organization for Standardization (ISO) https://www.iso.org/obp/ui/#search;
-American National Standards Institute (ANSI) https://www.ansi.org/;
-European Committee for Standardisation (CEN) https://www.cen.eu/Pages/default.aspx;
-British Standards Institute (BSI) https://www.bsigroup.com/en-GB/;
-TechStreet http://www.techstreet.com/
-IEEE Xplore Digital Library http://ieeexplore.ieee.org/Xplore/guesthome.jsp;

Furthermore, we will search data bases from regulatory and professional organisations for standards on health apps, medical devices, clinical information systems, and medicines:
-US FDA databases https://www.fda.gov/default.htm;
-European Medicines Agency (EMA) http://www.ema.europa.eu/ema/;
-European Commission https://ec.europa.eu/info/index_en;
-UK MHRA https://www.gov.uk/government/organisations/medicines-and-healthcare-products-regulatory-agency;
-The International Council for Harmonisation of Technical Requirements for Pharmaceuticals for Human Use (ICH): http://www.ich.org/;
-International Society for Pharmaceutical Engineering: https://www.ispe.org/;
-IEEE Computer Society: https://www.computer.org/;
-Advanced Safety in Health Technology: http://www.aami.org/;
-UK National Health Services (NHS) Digital: http://content.digital.nhs.uk/isce/publication/standards;
-Apple app store: https://developer.apple.com/app-store/guidelines/;
-Android app store: https://developer.android.com/distribute/best-practices/launch/launch-checklist.html.

For peer review only - http://bmjopen.bmj.com/site/about/guidelines.xhtml

Additionally, relevant articles on guidance, frameworks, and best practices for the development of health apps will be identified by searching the following electronic databases:
-MEDLINE through Ovid;
-EMBASE through Ovid;
-SCOPUS;
-ProQuest Technology Collection and Engineering Index (Compendex).

Search strategy

Preliminary draft search strategies for a regulatory website and MEDLINE can be found in the Supplementary File and will be further developed and tailored to the different databases. We will use the titles, abstracts and keywords of a set of articles for which we know that meet our inclusion criteria to define a search strategy that will return all these articles without an unmanageably large number of irrelevant articles. Also, we will hand-search reference lists and ask experts in the field to identify relevant standards.

Study records

Selection of studies

All search results will be imported into Zotero reference management software. We will exclude duplicate references by comparing titles, authors and digital object identifiers (DOIs) between similar search results. One reviewer will screen all titles and abstracts of search results independently against the inclusion and exclusion criteria. The second reviewer will screen 10% of these citations to validate the screening process. In case of high disagreement (>10%) the second reviewer will screen all citations. In case of multiple versions of a document the most recent and most broadly applicable geographically will be selected (i.e. the ISO international standard rather than the CEN European standard). One reviewer will retrieve full-text papers. When a full-text paper cannot be obtained, the authors will be contacted with a request to provide the publication. If no response is received, up to two attempts to contact the authors will be made. Two reviewers will assess full-text for eligibility, with any disagreement to be resolved through discussion with a third author. Selection of studies will be reported in a flow chart.

Data extraction & management

To extract data from included papers, one reviewer will use a standardized Excel form to extract data from included publications (see draft data extraction sheet in the Supplementary File). A second reviewer will validate data extraction by comparing the data extraction sheet with the original publication.

Data items

The data extraction form will be based on the Reporting Tool for Practice Guidelines in Health Care (the RIGHT Statement[20]) and include basic information (e.g. title, year published, focus), background (e.g. problem, aim, end-users), evidence (questions,

For peer review only

For peer review only - http://bmjopen.bmj.com/site/about/guidelines.xhtml

use of systematic reviews), recommendations/requirements (e.g. rationale), review and quality assurance, funding, declaration and management of interest, and other information (see the Supplementary File). The criteria have been adapted to make them relevant to health app development. Quality appraisal will be undertaken by assessing the proportion of items in the adapted RIGHT Statement[20] that are reported in the standards, guidelines, frameworks, and best practices.

Outcomes and prioritization

The primary outcome is to evaluate and determine the current standards for health app development. Secondary outcomes are to: 1) compare US and EU standards; 2) identify potential limitations in standards based on other software specific standards; 3) find opportunities to improve existing standards (e.g. patient safety, support innovation); and 4) determine and prioritize app development areas for focus in standards development.

Data synthesis

We will provide a narrative overview of findings and tabular summaries of extracted data. Also, we will analyze the relationship between different standards. Quantitative synthesis is inappropriate for the outcomes of this systematic review. This means that also no assessment of meta-biases and strength of the body of evidence will be undertaken.

Ethics and dissemination

No ethics approval is required. This review will systematically identify and assess standards, guidelines, frameworks, and best practices relevant for the development of health apps. The full systematic review will be submitted for publication in a peer-reviewed medical journal. A possible limitation of this review is that it only focuses on standards reported by international, US, European, and UK organizations; however, these are the markets of primary interest to the majority of app developers currently. The review will inform efforts that aim to improve the quality of health apps disseminated through existing links with relevant organisations, such as the BSI, Academic Health Sciences Network (AHSN), NHS Digital, National Institute for Health and Care Excellence (NICE), MHRA, Digital Health and Care Alliance (DHACA), Digital Health Oxford and London, and US FDA. This evaluation is a critical 'stepping stone' for future work to producing actionable guidelines for developers and adopters.

For peer review only - http://bmjopen.bmj.com/site/about/guidelines.xhtml

References

1. Ventola, C. L. Mobile Devices and Apps for Health Care Professionals: Uses and Benefits. *Pharm. Ther.* **39,** 356–364 (2014).
2. Research2Guidance. 325,000 mobile health apps available in 2017 – Android now the leading mHealth platform. (2017). Available at: https://research2guidance.com/325000-mobile-health-apps-available-in-2017/. (Accessed: 9th February 2018)
3. FDA. Mobile Medical Applications. Available at: https://www.fda.gov/MedicalDevices/DigitalHealth/MobileMedicalApplications/default.htm#a. (Accessed: 23rd January 2018)
4. Larsen, M. E., Nicholas, J. & Christensen, H. A Systematic Assessment of Smartphone Tools for Suicide Prevention. *PLOS ONE* **11,** e0152285 (2016).
5. Lewis, L. T. & Wyatt, C. J. mHealth and Mobile Medical Apps: A Framework to Assess Risk and Promote Safer Use. *J Med Internet Res* **16,** e210 (2014).
6. Nilsen, W. *et al.* Advancing the science of mHealth. *J. Health Commun.* **17 Suppl 1,** 5–10 (2012).
7. Champagne, D., Hung, A. & Leclerc, O. How pharma can win in a digital world.
8. ISO website. Available at: https://www.iso.org/standards.html. (Accessed: 16th February 2018)
9. European Medicines Agency (EMA) Quality guidelines. Available at: http://www.ema.europa.eu/ema/index.jsp?curl=pages/regulation/general/general_content_000081.jsp.

For peer review only - http://bmjopen.bmj.com/site/about/guidelines.xhtml

10. ISO 14971:2007 Medical devices -- Application of risk management to medical devices. Available at: https://www.iso.org/standard/38193.html.

11. International Society for Pharmaceutical Engineering ISPE's Good Automated Manufacturing Practice (GAMP) V-model. Available at: https://www.ispe.org/publications/guidance-documents/gamp-5. (Accessed: 23rd January 2018)

12. Tait, J. & Banda, G. Proportionate and adaptive governance of innovative technologies - The role of regulations, guidelines and standards. Available at: https://www.bsigroup.com/LocalFiles/en-GB/BIS/Innovate%20UK%20and%20emerging%20technologies/Summary%20Report%20-%20Adaptive%20governance%20-%20WEB.pdf. (Accessed: 16th February 2018)

13. BSI. How standards benefit businesses and the UK economy. Available at: https://www.bsigroup.com/LocalFiles/en-GB/standards/BSI-standards-brochure-how-standards-benefit-businesses-and-the-UK-economy-UK-EN.pdf. (Accessed: 23rd January 2018)

14. BSI. PAS 277:2015 Health and wellness apps. Quality criteria across the life cycle. Code of practice. Available at: https://shop.bsigroup.com/forms/PASs/PAS-2772015/. (Accessed: 23rd January 2018)

15. Association for the Advancement of Medical Instrumentation (AAMI) TIR45:2012 guidance. Available at: https://my.aami.org/store/detail.aspx?id=TIR45-PDF. (Accessed: 16th February 2018)

For peer review only - http://bmjopen.bmj.com/site/about/guidelines.xhtml

16. Shamseer, L. *et al.* Preferred reporting items for systematic review and meta-analysis protocols (PRISMA-P) 2015: elaboration and explanation. *BMJ* **349,** (2015).

17. Framework. (2018). Available at: https://techterms.com/definition/framework.

18. MHRA. Guidance - Medical devices: software applications (apps). Available at: https://www.gov.uk/government/publications/medical-devices-software-applications-apps. (Accessed: 2nd January 2018)

19. How to find standards in the Library. Available at: https://www.imperial.ac.uk/admin-services/library/find-books-articles-and-more/standards/.

20. Chen Y, Yang K, Marušić A & et al. A reporting tool for practice guidelines in health care: The right statement. *Ann. Intern. Med.* **166,** 128–132 (2017).

For peer review only

Authors' contributions

Michelle van Velthoven wrote the protocol. James Smith, David Brindley, and Glenn Wells provided substantial comments for important intellectual input on the protocol.

Funding statement

MHV is a Sir David Cooksey Fellow in Healthcare Translation at the University of Oxford and received no specific additional funding for this work. JS is supported by a UK Medical Research Council Studentship. The funder had no role in writing this protocol.

Competing interests statement

The author(s) declared the following potential competing interests with respect to the research, authorship, and/or publication of this article:
This article represents the authors' individual opinions and may not necessarily represent the viewpoints of their employers. MHV is the director of Dutches Consulting Ltd., which provides digital health-related advice to clients in the life sciences. DB is a stockholder in Translation Ventures Ltd. (Charlbury, Oxfordshire, UK) and IP Asset Ventures Ltd. (Oxford, Oxfordshire, UK), companies that, among other services, provide cell therapy biomanufacturing, regulatory, and financial advice to pharmaceutical clients. DB is also subject to the CFA Institute's codes, standards, and guidelines, so he must stress that this piece is provided for academic interest only and must not be construed in any way as an investment recommendation. Additionally, at the time of publication, DB and the organizations with which he is affiliated may or may not have agreed and/or have pending funding commitments from the organizations named here.

Patient and Public Involvement

Patients and the public were not involved in writing this protocol.

For peer review only

Supplementary File

Draft preliminary search

Regulatory website (International Organization for Standardization [ISO]) search terms (with limited search functions) in the technical sector of health (to be adapted for other websites)

1. Health app
2. Medical app
3. Health software
4. Medical software
5. Medical device
6. Health Information Technology System
7. Medical Information Technology System
8. Health IT system
9. Medical IT system

MEDLINE search strategy (to be adapted for other databases)

Health apps terms combined with OR:
Medical device*.ti,ab,kw.
Health app*.ti,ab,kw.
Medical app*.ti,ab,kw.
Wearable*.ti,ab,kw.
Mobile Applications/
Medical Informatics Applications/
Standards terms combined with OR:
(Standard* or framework* or Guideline* or Guidance or best practice* or Risk Assessment* or road map* or roadmap*) adj3 (Medical device* OR Health app* OR Medical app* OR Wearable*).ti,ab,kw.
Medical Device Legislation/st, td [Standards, Trends]
Device Approval/lj, st [Legislation & Jurisprudence, Standards]
Practice Guideline/
Risk Assessment/lj, mt, st [Legislation & Jurisprudence, Methods, Standards]
Government Regulation/

Final result = 1 AND 2

No limitations to be applied to the search strategy

Draft data extraction sheet

Items	Data	RIGHT checklist item[20]
Basic information		
Title/subtitle		1a
Year of publication		1b
Focus		1c
Scope		
Category (health app, medical device, software for medicine)		
Executive summary		2
Abbreviations and acronyms		3
Definitions		
Corresponding author/developer		4
Organisation		
Background		
Description of the problem		5
Aim		6
Primary population		7a
Subgroups		7b
Intended primary users		8a
Setting for intended use		8b
Document development group contributors		9a
Individuals involved in developing document		9b

For peer review only

Evidence		
Key questions		10a
Selection of outcomes		10b
Systematic review undertaken or used		11a
If based on existing systematic review, description of identification and assessment (provide the search strategies and the selection criteria, and description of how the risk of bias was evaluated) and whether they were updated		11b
Approach used for assessing evidence		12
Mention of other standards, guidelines, frameworks, and best practices		
Recommendations/requirements		
Clear, precise, and actionable recommendations/requirements		13a
Evidence for important subgroups		13b
Strength of recommendations/requirements		13c
Rationale/explanation for recommendations/requirements of target populations		14a
Cost and resource implication for recommendations/requirements		14b
Other factors taken into consideration when formulating the recommendations, such as equity, feasibility and acceptability		14c
Evidence to decision process		15
Review and quality assurance		
External review		16
Quality assurance		17

For peer review only

Funding, declaration and management of interest		
Sources of funding for all stages of document development		18a
Role of funder(s) in the different stages of document development and in the dissemination and implementation of the recommendations		18b
Types of conflicts (financial and non-financial)		19a
How conflicts of interest were evaluated and managed		19b
Other information		
Access		20
Suggestions for further research		21
Limitations of document		22
Comments		

For peer review only

Reporting checklist for protocol of a systematic review

Based on the PRISMA-P guidelines

Moher D, Shamseer L, Clarke M, Ghersi D, Liberati A, Petticrew M, Shekelle P, Stewart LA. Preferred Reporting Items for Systematic Review and Meta-Analysis Protocols (PRISMA-P) 2015 statement. Syst Rev. 2015;4(1):1.

		Reporting Item	Page Number
Identification	#1a	Identify the report as a protocol of a systematic review	1
Update	#1b	If the protocol is for an update of a previous systematic review, identify as such	na
	#2	If registered, provide the name of the registry (such as PROSPERO) and registration number	na
Contact	#3a	Provide name, institutional affiliation, e-mail address of all protocol authors; provide physical mailing address of corresponding author	1
Contribution	#3b	Describe contributions of protocol authors and identify the guarantor of the review	12
	#4	If the protocol represents an amendment of a previously completed or published protocol, identify as such and list changes; otherwise, state plan for documenting important protocol amendments	na
Sources	#5a	Indicate sources of financial or other support for the review	12
Sponsor	#5b	Provide name for the review funder and / or sponsor	12
Role of sponsor or funder	#5c	Describe roles of funder(s), sponsor(s), and / or institution(s), if any, in developing the protocol	12
Rationale	#6	Describe the rationale for the review in the context of what is already known	3-4
Objectives	#7	Provide an explicit statement of the question(s) the review will	5

For peer review only

		address with reference to participants, interventions, comparators, and outcomes (PICO)	
Eligibility criteria	#8	Specify the study characteristics (such as PICO, study design, setting, time frame) and report characteristics (such as years considered, language, publication status) to be used as criteria for eligibility for the review	5-6
Information sources	#9	Describe all intended information sources (such as electronic databases, contact with study authors, trial registers or other grey literature sources) with planned dates of coverage	6-7
Search strategy	#10	Present draft of search strategy to be used for at least one electronic database, including planned limits, such that it could be repeated	7+13
Study records - data management	#11a	Describe the mechanism(s) that will be used to manage records and data throughout the review	7
Study records - selection process	#11b	State the process that will be used for selecting studies (such as two independent reviewers) through each phase of the review (that is, screening, eligibility and inclusion in meta-analysis)	7
Study records - data collection process	#11c	Describe planned method of extracting data from reports (such as piloting forms, done independently, in duplicate), any processes for obtaining and confirming data from investigators	7
Data items	#12	List and define all variables for which data will be sought (such as PICO items, funding sources), any pre-planned data assumptions and simplifications	7-8
Outcomes and prioritization	#13	List and define all outcomes for which data will be sought, including prioritization of main and additional outcomes, with rationale	8
Risk of bias in individual studies	#14	Describe anticipated methods for assessing risk of bias of individual studies, including whether this will be done at the outcome or study level, or both; state how this information will be used in data synthesis	8
Data synthesis	#15a	Describe criteria under which study data will be quantitatively synthesised	8
	#15b	If data are appropriate for quantitative synthesis, describe	na

For peer review only

		planned summary measures, methods of handling data and methods of combining data from studies, including any planned exploration of consistency (such as I2, Kendall's τ)	
	#15c	Describe any proposed additional analyses (such as sensitivity or subgroup analyses, meta-regression)	na
	#15d	If quantitative synthesis is not appropriate, describe the type of summary planned	8
Meta-bias(es)	#16	Specify any planned assessment of meta-bias(es) (such as publication bias across studies, selective reporting within studies)	na
Confidence in cumulative evidence	#17	Describe how the strength of the body of evidence will be assessed (such as GRADE)	na

The PRISMA-P checklist is distributed under the terms of the Creative Commons Attribution License CC-BY 4.0. This checklist was completed on 13. March 2018 using http://www.goodreports.org/, a tool made by the EQUATOR Network in collaboration with Penelope.ai

For peer review only

BMJ Open

Digital health app development standards: a systematic review protocol

Journal:	*BMJ Open*
Manuscript ID	bmjopen-2018-022969.R1
Article Type:	Protocol
Date Submitted by the Author:	27-Jun-2018
Complete List of Authors:	Van Velthoven, Michelle; University of Oxford, Department of Paediatrics<br>Smith, James; University of Oxford, The Oxford – UCL Centre for the Advancement of Sustainable Medical Innovation (CASMI) Oxford; University of Oxford, Nuffield Department of Orthopaedics, Rheumatology and Musculoskeletal Sciences<br>Wells, Glenn; Oxford Academic Health Science Centre<br>Brindley, David; University of Oxford, Department of Paediatrics
**Primary Subject Heading**:	Health informatics
Secondary Subject Heading:	Research methods, Health policy
Keywords:	Health & safety < HEALTH SERVICES ADMINISTRATION & MANAGEMENT, Health policy < HEALTH SERVICES ADMINISTRATION & MANAGEMENT, Protocols & guidelines < HEALTH SERVICES ADMINISTRATION & MANAGEMENT, Quality in health care < HEALTH SERVICES ADMINISTRATION & MANAGEMENT, Telemedicine < BIOTECHNOLOGY & BIOINFORMATICS

SCHOLARONE™
Manuscripts

Digital health app development standards: a systematic review protocol

Michelle Helena van Velthoven[1], James Smith[2,3], Glenn Wells[4], David Brindley[1]

[1]Healthcare Translation Research Group, Department of Paediatrics, University of Oxford, Oxford, UK
[2]The Oxford – UCL Centre for the Advancement of Sustainable Medical Innovation (CASMI), The University of Oxford, New Richards Building, Old Road Campus, Oxford, OX3 7LG, UK
[3]Nuffield Department of Orthopaedics, Rheumatology and Musculoskeletal Sciences, University of Oxford, Old Road, Oxford, OX3 7LD, UK.
[4]Oxford Academic Health Sciences Centre, Oxford, UK

Corresponding author:
Michelle Helena van Velthoven
Michelle.vanvelthoven@paediatrics.ox.ac.uk
Mobile: 07425167828

Word count: 2268

For peer review only - http://bmjopen.bmj.com/site/about/guidelines.xhtml

Abstract (word count: 289)

Introduction

There is currently a lack of clear and accepted standards for the development (planning, requirement analysis and research, design and application testing) of apps for medical and healthcare use, which poses different risks to developers, providers, patients and the public. The aim of this work is to provide an overview of the current standards, frameworks, best practices and guidelines for the development of digital health apps. This review is a critical 'stepping stone' for further work on producing appropriate standards that can help mitigate risks (e.g. clinical, privacy and economic risks).

Methods and analysis

A systematic review identifying criteria from applicable standards, guidelines, frameworks, and best practices for the development of health apps. We will draw from standards for software for medical devices, clinical information systems, and medicine because of their relatedness and hope to apply lessons learned to apps. We will exclude other types of publications, and those published in languages other than English. We will search websites of relevant regulatory and professionals organisations. For health apps, we will also search electronic research databases (e.g. MEDLINE, EMBASE, SCOPUS, ProQuest Technology Collection and Engineering Index) because relevant publications may not be found on other websites. We will hand-search reference lists of included publications. The review will focus on international, US, European, and UK standards because these are the markets of primary interest to the majority of app developers currently. We will provide a narrative overview of findings and tabular summaries of extracted data. Also, we will examine the relationship between different standards and compare US and EU standards.

Ethics and dissemination

No ethics approval is required. The review will be disseminated through peer-reviewed publications, conference presentations, and inform efforts that aim to improve the quality of health apps through existing links with relevant organisations.

Key words: digital health, health apps, medical device, clinical software, medication

For peer review only - http://bmjopen.bmj.com/site/about/guidelines.xhtml

Strengths and limitations of this study

- This review will provide a systematic overview of standards for the development of health apps based on those for software of medical devices, clinical information systems, and medication given their relatedness.
- A comprehensive search of standards will be conducted.
- A limitation of this review is that it only focuses on standards reported by international organisations and those in the US, EU, and UK.
- The review will inform efforts that aim to improve the quality of health apps and is a critical 'stepping stone' to producing actionable guidelines for developers and adopters.

Introduction

Description of the issue

There is a lot of 'apptimism' for the potential of health apps to improve the quality of care and reduce costs.[1] However, despite a rapid growth of the health apps market with an estimated 325,000 health apps available in 2017[2] this potential has not been achieved. Health apps are software programs that are used in the context of healthcare on mobile communication devices, such as smartphones and tablets, that can also be used as accessories, such as wearable devices, or as a combination of accessories and software.[3] However, there are many low-quality and unsafe health apps and even apps with potentially harmful content.[4] This situation is resulting in different types of risks for users such as embarrassment, stigma, discrimination, stress, dissatisfaction, delay in effective treatment, poor lifestyle choices and deterioration in health.[5] Also, providers can be negatively impacted by reputation loss, poor quality of care, increase in undue demand on services, and opportunity losses.[5]

One of the reasons for the large number of low-quality health apps is that there are no agreed standards for their development, assessment, and appraisal. Health apps can be developed quickly, at any place and time by anyone interested, including people with non-medical backgrounds, which can create conflicting views on rapid technology development versus thorough evidence-based medicine principles.[6] Apps are often developed by start-ups with limited resources for research and development, which may result in short duration pilots with small participant numbers. Traditional healthcare companies with larger financial resources, such as pharmaceutical companies, on the other hand, have realized that they need to engage with digital health but are struggling given the differences between the development of drugs and digital tools.[7] As a result, there is a lack of consistency in the development of health apps.

Description of standards

A standard can be defined as "a document that provides requirements, specifications, guidelines or characteristics that can be used consistently to ensure that materials, products, processes, and services are fit for their purpose".[8] Standards are collaborative efforts, written by committees of manufacturers, users, research organizations, government departments and consumers.

For peer review only - http://bmjopen.bmj.com/site/about/guidelines.xhtml

Medical devices, clinical software and medicines have many standards, regulations and guidances for their development.[9] For example, the International Organization for Standardization (ISO) has a standard on software for medical devices, IEC 62304:2006 ' Medical device software - Software life cycle processes', which complements the main standard for medical devices, ISO 13485:2016 'Medical devices - Quality management systems - Requirements for regulatory purposes' and ISO 14971:2007, 'Application of risk management to medical devices'.[10] Similarly, in pharmaceutical manufacturing, standards exist such as International Society for Pharmaceutical Engineering (ISPE) Good Automated Manufacturing Practice (GAMP) for computerized systems[11], and are widely adopted.

However, for health software development there is the concern that standards will inhibit innovation. There needs to be a balance between basic principles for safe and efficient development of health apps that allows products to be built correctly and efficiently. Efforts have been made to develop more proportionate and adaptive governance of innovative technologies for different types of innovation, in different industries sectors.[12]

The benefits of standards for health apps

Standards can mitigate the risks of health apps, including clinical, privacy and economic risks, which are influenced by the function(s) of the health app, user and contextual factors.[5] Health apps are clinical software and can be divided into higher-risk apps classified as medical devices, such as clinical-decision-support apps, and lower-risk apps that are not, such as wellness and fitness apps.

Standards can help with developing appropriate products that are fit for purpose. Standards can have economic benefits such as contributing to the growth of economies, productivity and GDP, and exports.[13] For companies, using standards can also enhance their reputation; improve compliance with regulations; and encourage innovation through the diffusion of knowledge. For users, standards can ensure the safety, quality, and consistency of products.[13]

Why it is important to do this review

Previous efforts have developed standards for certain health apps, such as the British Standards Institute (BSI) PASS 277:2015, a standard for quality criteria for health and wellness apps across the life cycle[14] which builds on more established approaches for clinical software such as the Association for the Advancement of Medical Instrumentation (AAMI) TIR45:2012 guidance on the use of agile practices in the development of medical device software.[15] However, such guidance is focused specifically on the UK, and there is a clear need to provide an overview of standards applicable to all health apps across broader jurisdictions. Additionally, understanding and collating the requirements for software development in closely related fields would be useful in informing development of standards at a later date. We will conduct a systematic review to address these needs.

For peer review only - http://bmjopen.bmj.com/site/about/guidelines.xhtml

Objectives

This systematic review is part of a larger project that addresses the current lack of clear standards for apps for medical and healthcare use and the risk that not having these standards poses to developers, providers, patients and the public. The objectives of this systematic review are to:

1. Provide an overview of currently applicable standards, guidelines, frameworks, and best practices relevant for the development of digital health apps;
2. Look at other not directly applicable but related standards to see if relevant lessons can be learned from current software-specific guidance for medical devices, medication, and clinical information systems.

The review will inform efforts that aim to improve the quality of health apps and is a critical 'stepping stone' to further research on producing actionable guidelines for developers and adopters.

Methods and analysis

This is the protocol for a systematic review that is reported where possible according to the Preferred Reporting Items for Systematic Reviews and Meta-Analyses for protocols (PRISMA-P),[16] which is provided as a supporting document.

Patient and Public Involvement

Patients and the public were not involved in writing this protocol.

Criteria for considering publications

We will include applicable standards, guidelines, frameworks, and best practices for the development (planning, requirement analysis and research, design and application testing[14]) of health apps. We will draw from software standards for medical devices, clinical information systems, and medicine because of their relatedness and apply hope to apply lessons learnt to apps.

Standards are requirements, specifications, guidelines or characteristics that can be used consistently to ensure that materials, products, processes, and services are fit for their purpose. Guidelines are advice or information aimed at resolving a problem or difficulty while frameworks are underlying structures for describing a process. A framework is 'a platform for developing software applications. It provides a foundation on which software developers can build programs for a specific platform.'[17] Best practice is a method or technique that has been generally accepted as superior to any alternatives.

An app is defined similarly by different organisations,[3,18] for example by the US Food and Drug Administration (FDA) as "software programs that run on smartphones and other mobile communication devices. They can also be accessories that attach to a smartphone or other mobile communication devices, or a combination of accessories and software".[3] In the context of healthcare, the FDA defines mobile medical apps as 'medical devices that are mobile apps, meet the

For peer review only - http://bmjopen.bmj.com/site/about/guidelines.xhtml

definition of a medical device and are an accessory to a regulated medical device or transform a mobile platform into a regulated medical device'.[3] The Medicines and Health Regulatory Authority (MHRA) broadly considers health apps to be medical devices if they have a medical purpose (e.g. prevention, diagnosis, monitoring, treatment of disease, diagnosis of disease, injury or handicap, compensation for injury or handicap, investigation, replacement of modification of the anatomy or of a physiological process, control of conception).[18] The BSI considers a health or wellness app when it 'contributes to any aspect of the physical, mental or social wellbeing of the user or any other subject of care or wellbeing'.[14]

We will exclude other types of papers, such as editorials, opinion pieces, viewpoints, and publications in languages other than English. It will not be possible to provide an overview of standards in all countries around the world given our limited resources. Therefore, we will focus on international, US, European, and UK standards because these are the markets of primary interest to the majority of app developers currently.

Information sources

We will search the following standards databases for health apps, medical devices, clinical software, and medicines advised by Imperial College London librarians (2007 till date of search)[19]:
-International Organization for Standardization (ISO) https://www.iso.org/obp/ui/#search;
-American National Standards Institute (ANSI) https://www.ansi.org/;
-European Committee for Standardisation (CEN) https://www.cen.eu/Pages/default.aspx;
-British Standards Institute (BSI) https://www.bsigroup.com/en-GB/;
-TechStreet http://www.techstreet.com/
-IEEE Xplore Digital Library http://ieeexplore.ieee.org/Xplore/guesthome.jsp;

Furthermore, we will search databases from regulatory and professional organisations for standards on health apps, medical devices, clinical information systems, and medicines (2007 till date of search):
-US FDA databases https://www.fda.gov/default.htm;
-European Medicines Agency (EMA) http://www.ema.europa.eu/ema/;
-European Commission https://ec.europa.eu/info/index_en;
-UK MHRA https://www.gov.uk/government/organisations/medicines-and-healthcare-products-regulatory-agency;
-The International Council for Harmonisation of Technical Requirements for Pharmaceuticals for Human Use (ICH): http://www.ich.org/;
-International Society for Pharmaceutical Engineering: https://www.ispe.org/;
-IEEE Computer Society: https://www.computer.org/;
-Advanced Safety in Health Technology: http://www.aami.org/;
-UK National Health Services (NHS) Digital: http://content.digital.nhs.uk/isce/publication/standards;
-Apple app store: https://developer.apple.com/app-store/guidelines/;

For peer review only - http://bmjopen.bmj.com/site/about/guidelines.xhtml

-Android app store: https://developer.android.com/distribute/best-practices/launch/launch-checklist.html.

Additionally, relevant articles on guidance, frameworks, and best practices for the development of health apps will be identified by searching the following electronic databases (2007 till date of search):
-MEDLINE through Ovid;
-EMBASE through Ovid;
-SCOPUS;
-ProQuest Technology Collection and Engineering Index (Compendex).

Search strategy

Preliminary draft search strategies for a regulatory website and MEDLINE can be found in the Supplementary File and will be further developed and tailored to the different databases. We will use the titles, abstracts and keywords of a set of articles for which we know that meet our inclusion criteria to define a search strategy that will return all these articles without an unmanageably large number of irrelevant articles. Also, we will hand-search reference lists and ask experts in the field to identify relevant standards.

Study records

Selection of studies

All search results will be imported into Zotero reference management software. We will exclude duplicate references by comparing titles, authors and digital object identifiers (DOIs) between similar search results. One reviewer will screen all titles and abstracts of search results independently against the inclusion and exclusion criteria. The second reviewer will screen 10% of these citations to validate the screening process. In case of high disagreement (>10%) the second reviewer will screen all citations. In case of multiple versions of a document the most recent and most broadly applicable geographically will be selected (i.e. the ISO international standard rather than the CEN European standard). One reviewer will retrieve full-text papers. When a full-text paper cannot be obtained, the authors will be contacted with a request to provide the publication. If no response is received, up to two attempts to contact the authors will be made. Two reviewers will assess full-text for eligibility, with any disagreement to be resolved through discussion with a third author. Selection of studies will be reported in a flow chart.

Data extraction & management

To extract data from included papers, one reviewer will use a standardized Excel form to extract data from included publications (see draft data extraction sheet in the Supplementary File). A second reviewer will validate data extraction by comparing the data extraction sheet with the original publication.

For peer review only - http://bmjopen.bmj.com/site/about/guidelines.xhtml

Data items

The data extraction form will be based on the Reporting Tool for Practice Guidelines in Health Care (the RIGHT Statement[20]) and include basic information (e.g. title, year published, focus), background (e.g. problem, aim, end-users), evidence (questions, use of systematic reviews), recommendations/requirements (e.g. rationale), review and quality assurance, funding, declaration and management of interest, and other information (see the Supplementary File). The criteria have been adapted to make them relevant to health app development. Quality appraisal will be undertaken by assessing the proportion of items in the adapted RIGHT Statement[20] that are reported in the standards, guidelines, frameworks, and best practices.

Outcomes and prioritization

The primary outcome is to evaluate and determine the current standards for health app development. Secondary outcomes are to: 1) compare US and EU standards; 2) identify potential limitations in standards based on other software specific standards; 3) find opportunities to improve existing standards (e.g. patient safety, support innovation); and 4) determine and prioritize app development areas for focus in standards development.

Data synthesis

We will provide a narrative overview of findings and tabular summaries of extracted data. Also, we will analyze the relationship between different standards. Quantitative synthesis is inappropriate for the outcomes of this systematic review. This means that also no assessment of meta-biases and strength of the body of evidence will be undertaken.

Ethics and dissemination

No ethics approval is required. This review will systematically identify and assess standards, guidelines, frameworks, and best practices relevant for the development of health apps. The full systematic review will be submitted for publication in a peer-reviewed medical journal. A possible limitation of this review is that it only focuses on standards reported by international, US, European, and UK organizations; however, these are the markets of primary interest to the majority of app developers currently. The review will inform efforts that aim to improve the quality of health apps disseminated through existing links with relevant organisations, such as the BSI, Academic Health Sciences Network (AHSN), NHS Digital, National Institute for Health and Care Excellence (NICE), MHRA, Digital Health and Care Alliance (DHACA), Digital Health Oxford and London, and US FDA. This evaluation is a critical 'stepping stone' for future work to producing actionable guidelines for developers and adopters.

For peer review only - http://bmjopen.bmj.com/site/about/guidelines.xhtml

For peer review only

For peer review only - http://bmjopen.bmj.com/site/about/guidelines.xhtml

References

1. Ventola, C. L. Mobile Devices and Apps for Health Care Professionals: Uses and Benefits. *Pharm. Ther.* **39,** 356–364 (2014).
2. Research2Guidance. 325,000 mobile health apps available in 2017 – Android now the leading mHealth platform. (2017). Available at: https://research2guidance.com/325000-mobile-health-apps-available-in-2017/. (Accessed: 9th February 2018)
3. FDA. Mobile Medical Applications. Available at: https://www.fda.gov/MedicalDevices/DigitalHealth/MobileMedicalApplications/default.htm#a. (Accessed: 23rd January 2018)
4. Larsen, M. E., Nicholas, J. & Christensen, H. A Systematic Assessment of Smartphone Tools for Suicide Prevention. *PLOS ONE* **11,** e0152285 (2016).
5. Lewis, T. L. & Wyatt, J. C. mHealth and Mobile Medical Apps: A Framework to Assess Risk and Promote Safer Use. *J Med Internet Res* **16,** e210 (2014).
6. Nilsen, W. *et al.* Advancing the science of mHealth. *J. Health Commun.* **17 Suppl 1,** 5–10 (2012).
7. Champagne, D., Hung, A. & Leclerc, O. How pharma can win in a digital world. Available at: https://www.mckinsey.com/industries/pharmaceuticals-and-medical-products/our-insights/how-pharma-can-win-in-a-digital-world. (Accessed: 11th April 2018)
8. ISO website. Available at: https://www.iso.org/standards.html. (Accessed: 16th February 2018)

For peer review only

For peer review only - http://bmjopen.bmj.com/site/about/guidelines.xhtml

9. European Medicines Agency (EMA) Quality guidelines. Available at: http://www.ema.europa.eu/ema/index.jsp?curl=pages/regulation/general/general_content_000081.jsp. (Accessed: 27th June 2018)

10. ISO 14971:2007 Medical devices -- Application of risk management to medical devices. Available at: https://www.iso.org/standard/38193.html.

11. International Society for Pharmaceutical Engineering ISPE's Good Automated Manufacturing Practice (GAMP) V-model. Available at: https://www.ispe.org/publications/guidance-documents/gamp-5. (Accessed: 23rd January 2018)

12. Tait, J. & Banda, G. Proportionate and adaptive governance of innovative technologies - The role of regulations, guidelines and standards. Available at: https://www.bsigroup.com/LocalFiles/en-GB/BIS/Innovate%20UK%20and%20emerging%20technologies/Summary%20Report%20-%20Adaptive%20governance%20-%20WEB.pdf. (Accessed: 16th February 2018)

13. BSI. How standards benefit businesses and the UK economy. Available at: https://www.bsigroup.com/LocalFiles/en-GB/standards/BSI-standards-brochure-how-standards-benefit-businesses-and-the-UK-economy-UK-EN.pdf. (Accessed: 23rd January 2018)

14. BSI. PAS 277:2015 Health and wellness apps. Quality criteria across the life cycle. Code of practice. Available at: https://shop.bsigroup.com/forms/PASs/PAS-2772015/. (Accessed: 23rd January 2018)

For peer review only - http://bmjopen.bmj.com/site/about/guidelines.xhtml

15. Association for the Advancement of Medical Instrumentation (AAMI) TIR45:2012 guidance. Available at: https://my.aami.org/store/detail.aspx?id=TIR45-PDF. (Accessed: 16th February 2018)

16. Shamseer, L. *et al.* Preferred reporting items for systematic review and meta-analysis protocols (PRISMA-P) 2015: elaboration and explanation. *BMJ* **349,** (2015).

17. Framework definition. Available at: https://techterms.com/definition/framework. (Accessed: 23rd February 2018)

18. MHRA. Guidance - Medical devices: software applications (apps). Available at: https://www.gov.uk/government/publications/medical-devices-software-applications-apps. (Accessed: 2nd January 2018)

19. How to find standards in the Library. Available at: https://www.imperial.ac.uk/admin-services/library/find-books-articles-and-more/standards/.

20. Chen Y, Yang K, Marušić A & et al. A reporting tool for practice guidelines in health care: The right statement. *Ann. Intern. Med.* **166,** 128–132 (2017).

For peer review only

For peer review only - http://bmjopen.bmj.com/site/about/guidelines.xhtml

Authors' contributions

Michelle van Velthoven wrote the protocol. James Smith, David Brindley, and Glenn Wells provided substantial comments for important intellectual input on the protocol.

Funding statement

MHV is a Sir David Cooksey Fellow in Healthcare Translation at the University of Oxford and received no specific additional funding for this work. JS is supported by a UK Medical Research Council Studentship. The funder had no role in writing this protocol.

Competing interests statement

The author(s) declared the following potential competing interests with respect to the research, authorship, and/or publication of this article:
This article represents the authors' individual opinions and may not necessarily represent the viewpoints of their employers. MHV is the director of Dutches Consulting Ltd., which provides digital health-related advice to clients in the life sciences. DB is a stockholder in Translation Ventures Ltd. (Charlbury, Oxfordshire, UK) and IP Asset Ventures Ltd. (Oxford, Oxfordshire, UK), companies that, among other services, provide cell therapy biomanufacturing, regulatory, and financial advice to pharmaceutical clients. DB is also subject to the CFA Institute's codes, standards, and guidelines, so he must stress that this piece is provided for academic interest only and must not be construed in any way as an investment recommendation. Additionally, at the time of publication, DB and the organizations with which he is affiliated may or may not have agreed and/or have pending funding commitments from the organizations named here.

For peer review only

For peer review only - http://bmjopen.bmj.com/site/about/guidelines.xhtml

Supplementary File

Draft preliminary search

<u>Regulatory website (International Organization for Standardization [ISO]) search terms (with limited search functions) in the technical sector of health (to be adapted for other websites)</u>

1. Health app
2. Medical app
3. Health software
4. Medical software
5. Medical device
6. Health Information Technology System
7. Medical Information Technology System
8. Health IT system
9. Medical IT system

<u>MEDLINE search strategy (to be adapted for other databases)</u>

Health apps terms combined with OR:
Medical device*.ti,ab,kw.
Health app*.ti,ab,kw.
Medical app*.ti,ab,kw.
Wearable*.ti,ab,kw.
Mobile Applications/
Medical Informatics Applications/
Standards terms combined with OR:
(Standard* or framework* or Guideline* or Guidance or best practice* or Risk Assessment* or road map* or roadmap*) adj3 (Medical device* OR Health app* OR Medical app* OR Wearable*).ti,ab,kw.
Medical Device Legislation/st, td [Standards, Trends]
Device Approval/lj, st [Legislation & Jurisprudence, Standards]
Practice Guideline/
Risk Assessment/lj, mt, st [Legislation & Jurisprudence, Methods, Standards]
Government Regulation/

Final result = 1 AND 2

No limitations to be applied to the search strategy

For peer review only

Draft data extraction sheet

**Items**	**Data**	**RIGHT checklist item**[20]
Basic information		
Title/subtitle		1a
Year of publication		1b
Focus		1c
Scope		
Category (health app, medical device, software for medicine)		
Executive summary		2
Abbreviations and acronyms		3
Definitions		
Corresponding author/developer		4
Organisation		
Background		
Description of the problem		5
Aim		6
Primary population		7a
Subgroups		7b
Intended primary users		8a
Setting for intended use		8b
Document development group contributors		9a
Individuals involved in developing document		9b

For peer review only

Evidence		
Key questions		10a
Selection of outcomes		10b
Systematic review undertaken or used		11a
If based on existing systematic review, description of identification and assessment (provide the search strategies and the selection criteria, and description of how the risk of bias was evaluated) and whether they were updated		11b
Approach used for assessing evidence		12
Mention of other standards, guidelines, frameworks, and best practices		
Recommendations/requirements		
Clear, precise, and actionable recommendations/requirements		13a
Evidence for important subgroups		13b
Strength of recommendations/requirements		13c
Rationale/explanation for recommendations/requirements of target populations		14a
Cost and resource implication for recommendations/requirements		14b
Other factors taken into consideration when formulating the recommendations, such as equity, feasibility and acceptability		14c
Evidence to decision process		15
Review and quality assurance		
External review		16
Quality assurance		17

For peer review only

Funding, declaration and management of interest		
Sources of funding for all stages of document development		18a
Role of funder(s) in the different stages of document development and in the dissemination and implementation of the recommendations		18b
Types of conflicts (financial and non-financial)		19a
How conflicts of interest were evaluated and managed		19b
Other information		
Access		20
Suggestions for further research		21
Limitations of document		22
Comments		

For peer review only

Reporting checklist for protocol of a systematic review

Based on the PRISMA-P guidelines

Moher D, Shamseer L, Clarke M, Ghersi D, Liberati A, Petticrew M, Shekelle P, Stewart LA. Preferred Reporting Items for Systematic Review and Meta-Analysis Protocols (PRISMA-P) 2015 statement. Syst Rev. 2015;4(1):1.

		Reporting Item	Page Number
Identification	#1a	Identify the report as a protocol of a systematic review	1
Update	#1b	If the protocol is for an update of a previous systematic review, identify as such	na
	#2	If registered, provide the name of the registry (such as PROSPERO) and registration number	na
Contact	#3a	Provide name, institutional affiliation, e-mail address of all protocol authors; provide physical mailing address of corresponding author	1
Contribution	#3b	Describe contributions of protocol authors and identify the guarantor of the review	12
	#4	If the protocol represents an amendment of a previously completed or published protocol, identify as such and list changes; otherwise, state plan for documenting important protocol amendments	na
Sources	#5a	Indicate sources of financial or other support for the review	12
Sponsor	#5b	Provide name for the review funder and / or sponsor	12
Role of sponsor or funder	#5c	Describe roles of funder(s), sponsor(s), and / or institution(s), if any, in developing the protocol	12
Rationale	#6	Describe the rationale for the review in the context of what is already known	3-4
Objectives	#7	Provide an explicit statement of the question(s) the review will	5

For peer review only

		address with reference to participants, interventions, comparators, and outcomes (PICO)	
Eligibility criteria	#8	Specify the study characteristics (such as PICO, study design, setting, time frame) and report characteristics (such as years considered, language, publication status) to be used as criteria for eligibility for the review	5-6
Information sources	#9	Describe all intended information sources (such as electronic databases, contact with study authors, trial registers or other grey literature sources) with planned dates of coverage	6-7
Search strategy	#10	Present draft of search strategy to be used for at least one electronic database, including planned limits, such that it could be repeated	7+13
Study records - data management	#11a	Describe the mechanism(s) that will be used to manage records and data throughout the review	7
Study records - selection process	#11b	State the process that will be used for selecting studies (such as two independent reviewers) through each phase of the review (that is, screening, eligibility and inclusion in meta-analysis)	7
Study records - data collection process	#11c	Describe planned method of extracting data from reports (such as piloting forms, done independently, in duplicate), any processes for obtaining and confirming data from investigators	7
Data items	#12	List and define all variables for which data will be sought (such as PICO items, funding sources), any pre-planned data assumptions and simplifications	7-8
Outcomes and prioritization	#13	List and define all outcomes for which data will be sought, including prioritization of main and additional outcomes, with rationale	8
Risk of bias in individual studies	#14	Describe anticipated methods for assessing risk of bias of individual studies, including whether this will be done at the outcome or study level, or both; state how this information will be used in data synthesis	8
Data synthesis	#15a	Describe criteria under which study data will be quantitatively synthesised	8
	#15b	If data are appropriate for quantitative synthesis, describe	na

For peer review only

		planned summary measures, methods of handling data and methods of combining data from studies, including any planned exploration of consistency (such as I2, Kendall's τ)	
	#15c	Describe any proposed additional analyses (such as sensitivity or subgroup analyses, meta-regression)	na
	#15d	If quantitative synthesis is not appropriate, describe the type of summary planned	8
Meta-bias(es)	#16	Specify any planned assessment of meta-bias(es) (such as publication bias across studies, selective reporting within studies)	na
Confidence in cumulative evidence	#17	Describe how the strength of the body of evidence will be assessed (such as GRADE)	na

The PRISMA-P checklist is distributed under the terms of the Creative Commons Attribution License CC-BY 4.0. This checklist was completed on 13. March 2018 using http://www.goodreports.org/, a tool made by the EQUATOR Network in collaboration with Penelope.ai

For peer review only
